# NTN1 regulates autophagy through the MAP1B/DAPK1 axis to ameliorate acute kidney injury *in vitro*

**DOI:** 10.1515/med-2025-1374

**Published:** 2026-05-19

**Authors:** Meng Zhang, Heyu Tian, Jin Lin, Xi Wu, Deyuan Zhi

**Affiliations:** Department of Critical Care Medicine, Beijing Friendship Hospital, Capital Medical University, Beijing, China

**Keywords:** sepsis-associated acute kidney injury, netrin 1, microtubule associated protein 1B, death associated protein kinase 1

## Abstract

**Objectives:**

Sepsis-associated acute kidney injury (S-AKI) is a common complication, while Netrin 1 (NTN1) is a marker of kidney injury-related diseases. This study thus investigated how NTN1 regulates S-AKI.

**Methods:**

HK-2 cells were exposed to lipopolysaccharides (LPS), where NTN1 expression was manipulated to study the effects of NTN1 on apoptosis, inflammatory factors (tumor necrosis factor-α (TNF-α), interleukin (IL)-1β and IL-6), and reactive oxygen species (ROS) levels. Western blot was conducted to assess the role of NTN1 in microtubule associated protein 1B (MAP1B) phosphorylation and autophagy marker protein. Cells were treated with NTN1 overexpression and MAP1B phosphorylation inhibitors to determine the connection of NTN1 and MAP1B. The interaction between MAP1B and DAPK1 was investigated by co-immunoprecipitation. Following the successful generation of DAPK1 knockdown and/or overexpressing cell models, ROS and membrane blebbing modifications were subsequently assessed.

**Results:**

Overexpression of NTN1 suppressed HK-2  cell apoptosis, inflammation, and ROS. MAP1B was an interacting protein of NTN1. NTN1 overexpression increased Atg5 protein expression and light chain 3 (LC3)II/LC3I levels in HK-2 cells, which was reversed by MAP1B phosphorylation inhibitors. MAP1B interacted with DAPK1. DAPK1 knockdown enhanced apoptosis, ROS, and membrane vesicle inhibition, which was offset by MAP1B overexpression.

**Conclusions:**

NTN1 facilitates cell membrane blebbing to induce autophagy via regulating MAP1B and DAPK1, thereby alleviating LPS-induced AKI *in vitro*.

## Highlights

1. NTN1 alleviates septic AKI injury by activating a novel MAP1B-DAPK1 signaling axis.
2. MAP1B phosphorylation plays a pivotal role in NTN1-mediated cytoprotection and autophagy promotion.
3. MAP1B promotes autophagy and mitigates tubular injury through DAPK1.

## Introduction

Acute kidney injury (AKI) refers to a rapid decline in kidney function in a short period of time, manifested as reduced urine output, edema, and disturbance of the internal environment [1], 2]. There are many established causes of AKI, such as insufficient renal perfusion, serious infection, renal toxic substances, and urinary tract obstruction [3]. Sepsis is a life-threatening disease triggered by pathogenic invasion that leads to organ dysfunction [4], and it commonly involves the kidneys, the organs responsible for glomerular filtration [5]. The overlap between sepsis and AKI is substantial: up to 50 % of AKI cases belong to sepsis-associated acute kidney injury (S-AKI), and up to 60 % of patients with sepsis also develop AKI [6]. Despite advances in medical science and updates in therapeutic routes, the incidence and mortality of S-AKI are still high, and little is known about its pathogenesis [7]. Notably, dysregulated immune-inflammatory responses and aberrant cell death processes (e.g., apoptosis, autophagy) are central to the pathophysiology of both sepsis and cancers, suggesting potential shared mechanistic nodes [8], [9], [10]. Thus, elucidating the pathogenesis of S-AKI will directly translate into more effective treatment strategies, contributing to shortening the hospital stay and improving survival for patients.

Netrin-1 (NTN1) is a diffusible laminin related protein that can regulate neuronal axon migration, angiogenesis, and tissue survival [11]. NTN1 is abnormally expressed in a variety of diseases, including diabetes, cardiovascular diseases and cancer [11], 12], and has been proved to be an important indicator for the diagnosis and prognosis of breast cancer [13], colorectal cancer [14], gastric cancer [15] and non-small cell lung cancer [16]. Moreover, in many cancers, upregulation of NTN1 is observed, and is considered as a selective mechanism that can prevent apoptosis induced by dependence receptors (deleted in colorectal cancer (DCC), uncoordinated-5 homolog (UNC5H)) [11], 17]. NTN1 is prominently localized in the interstitium in normal kidney, with expression consistent with that in peritubular capillaries [18]. Studies have suggested NTN1 as a marker in multiple kidney injury related diseases [19], [20], [21]. Of note, NTN1 has been shown to ameliorate renal injury by promoting tubular epithelial cell proliferation and inhibiting cell apoptosis [22], 23]. The urinary NTN1 level in patients with AKI is increased significantly, especially in the sepsis group, showing that NTN1 may serve as a new diagnostic index of early AKI [24]. But there are few reports about the specific regulatory mechanism of NTN1 in S-AKI.

Therefore, this study investigated the specific role and mechanism of NTN1 on S-AKI through lipopolysaccharides (LPS)-induced cell model, with a view to providing more comprehensive understanding of the pathogenesis of S-AKI.

## Materials and methods

### Bioinformatics assay

String website (https://string-db.org/) was used to analyze the interaction between microtubule associated protein 1B (MAP1B) and NTN1.

### Cell culture

Renal tubular epithelial cell line HK-2 (AW-CELLS-H0142, Anweisci, China), and RPTEC/TERT1 cells (CRL-4031, ATCC, Virginia, MA, USA) were placed in Minimum Essential Medium (MEM, BC-M-019, Sbjbio, China) enriched with 10 % fetal bovine serum (FBS, FCS500, ExCell, China) at 37 °C with 5 % CO_2_. The cells were identified by short tandem repeat upon receipt.

### Transfection

Overexpression plasmid NTN1 (OE-NTN1) or MAP1B (OE-MAP1B) was synthesized based on the pcDNA3.1 vector (VT9221, YouBia, China), and the empty vector acted as the negative control (OE-NC). The small interfering RNA (siRNA) targeting NTN1 (si-NTN1, AGC​TGA​AGA​TTA​ACA​TGA​AAA​AG) or death associated protein kinase 1 (si-DAPK1, AGG​AAA​ACG​TGG​ATG​ATT​ACT​AC) and their negative control (si-NC) of si-NTN1 or si-DAPK1 were purchasable from Genepharma (China). Next, cell transfection was accomplished by Lipofectamine 3,000 reagent (L3000-015, Invitrogen, USA). In brief, HK-2 or RPTEC/TERT1 cells were inoculated into 6-well plates (3 × 10^5^ cells/well) until reaching 80 % confluence. Thereafter, Lipofectamine 3,000 reagent and the overexpressed plasmids (2.5 μg) or siRNA (100 pmol) were separately diluted in serum-free MEM (125 μL) to form the mixtures, followed by the incubation of cells and mixtures. 48 h after transfection, quantitative real-time polymerase chain reaction (qRT-PCR) was applied to assess the transfection rate.

### Cell treatment and grouping

To assess the role of NTN1 in renal injury *in vitro*, HK-2 or RPTEC/TERT1 cells were transfected with NTN1 overexpression plasmid or si-NTN1 and then stimulated with 10 μg/mL lipopolysaccharide (LPS, HY-D1056, MedChemExpress, China) to construct cell injury model [25]. In this part, the groups are as follows: Blank group, OE-NC group, OE-NTN1 group, si-NC group and si-NTN1 group. In addition, to reveal the mechanism of NTN1 on LPS-induced HK-2 or RPTEC/TERT1 cells, the phosphorylation inhibitor U0126 (10 μM, S1102, Selleck, China) was added to treat HK-2 cells in the OE-NTN1 group to prevent the phosphorylation of MAP1B [26].

Next, to determine the interaction between MAP1B and DAPK1, si-DAPK1 and MAP1B overexpression plasmid were transfected into HK-2 cells, followed by the treatment of LPS. The groups in this part included: Control (CON) group, si-NC group, si-DAPK1 group, OE-NC group, OE-MAP1B group, si-NC+OE-NC group, si-DAPK1+OE-NC group, si-NC+OE-MAP1B group, and si-DAPK1+OE-MAP1B group.

Further, to verify the analysis of autophagy flux, bafilomycin (HY-100558, MedChemexpress, Monmouth Junction, NJ, USA) was used in this study. After transfection with si-DAPK1 or MAP1B overexpression plasmid, HK-2 cells were then treated with bafilomycin (400 nM) for 4 h [27].

**Figure 1:**
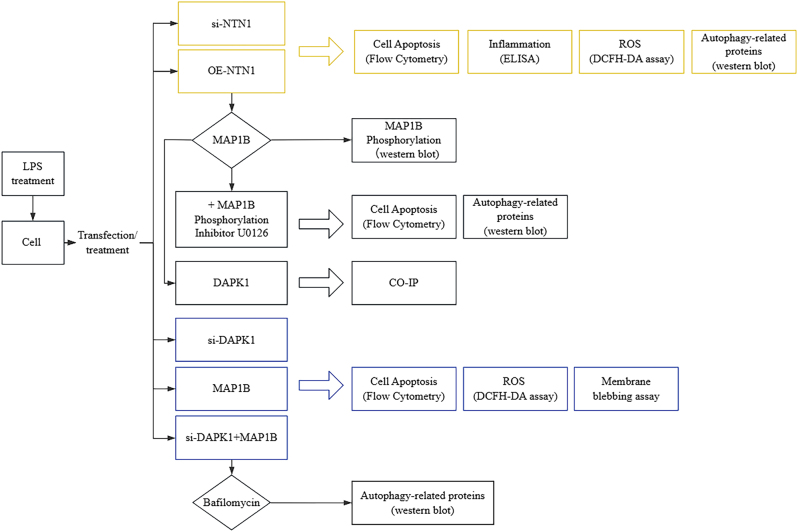
The experimental flow chart.

The experimental flow chart is shown in Figure 1.

### qRT-PCR

Total RNA from HK-2 or RPTEC/TERT1 cells was isolated with TRIzol reagent (15,596-026, Invitrogen, USA). Next, reverse transcription kit (D7178, Beyotime, China) was employed to synthesize cDNA. qRT-PCR was conducted on the PCR instrument (CFX96, Bio-Rad, USA) with universal PCR master mix (A46113, Applied Biosystems, USA). GAPDH was exploited as the housekeeping gene. The 2^−ΔΔCt^ method was adopted to quantify the expressions of mRNAs [28]. Primers are shown as follows (5′-3′): NTN1, (F) CTA​TGT​GGG​AGG​GAG​GGA​CA, (R) CAT​GGC​CCA​CAG​GAA​TGT​CT; DAPK1, (F) GCG​AGG​GCT​TCA​TTC​TTC​CG, (R) AGT​GGG​CGA​TTT​GAA​GGG​AG; MAP1B, (F) TGA​ATT​CCT​GGG​CAA​ACT​GGT, (R) CCA​CTC​GGT​CTA​AGT​GTC​GG; GAPDH, (F) GGT​CAC​CAG​GGC​TGC​TTT​TA, (R) CCC​GTT​CTC​AGC​CAT​GTA​GT.

### Flow cytometry assay

The flow cytometry assay was performed utilizing an Annexin V-FITC/PI apoptosis detection kit (C0007-1, Applygen, China). HK-2 or RPTEC/TERT1 cells (5 × 10^4^ per well) were inoculated into 6-well plates. After the transfection, cells were resuspended in 195 μL binding buffer, and then stained with 5 μL Annexin V-FITC and 10 μL propidium iodide (PI) at 37 °C for 15 min. At last, the signals of FITC and PI were detected using a flow cytometer (CytoFLEX, Beckman Coulter, USA). Apoptotic rate (%)=percentage of early apoptotic cells + percentage of late apoptotic cells.

### Enzyme-linked immunosorbent assay (ELISA)

Human ELISA kits for detecting the levels of tumor necrosis factor-α (TNF-α, PT518), interleukin (IL)-1β (PI305), and IL-6 (PI330) were purchased from Beyotime (China). According to the producer’s directions, the supernatants of HK-2 cells were diluted, incubated first with anti-TNF-α/IL-1β/IL-6 antibody in the 96-well plates at 37 °C for 1 h, and then with streptavidin-horseradish peroxidase (HRP) at 37 °C for 30 min. Afterwards, samples were reacted with color developing reagent for 20 min and then the reaction was ceased with stop solution. The absorbance (450 nm) was evaluated by a microplate reader (PLUS 384, Molecular Devices, USA).

### Reactive oxygen species (ROS) detection

The ROS level of HK-2 cells was determined using 2,7-Dichlorodi-hydrofluorescein diacetate (DCFH-DA) reagent (S0033S, Beyotime). In brief, DCFH-DA was diluted with serum-free medium at a ratio of 1:1,000 to reach a final concentration of 10 μmol/L. Next, cells were cultivated with DCFH-DA buffer for 20 min at 37 °C, followed by washing with phosphate buffered saline (PBS, B1201, Applygen, China) three times. Ultimately, the DCFH-DA fluorescence in cells was observed under the microscope (WMF-3590, Shanghai Wumo Optical Instrument Co., Ltd, China).

### Membrane blebbing assay

Membrane blebbing assay was performed as described in a prior study [29]. MAP1B overexpression plasmid and si-DAPK1 were co-transfected into HK-2 cells for 48 h, and then cells were fixed with 4 % paraformaldehyde (P6148, Sigma-Aldrich, USA) for 10 min. After washing with PBS, cells were incubated with the rabbit-anti MAP1B (1:200, ab224115, Abcam) and rabbit-anti DAPK1 (1:200, MA566875, Invitrogen, USA) for 1 h. Next, cells were stained with Alexa Fluor 488 conjugated goat anti-rabbit (ab150077, Abcam) prior to the observation with a fluorescent microscope (×400). Blebbing cells are defined as spherical protrusions formed on the plasma membrane. The proportion of blebbing cells (%) was counted as the blebbing cell number/the total cell number×100 %.

### Western blot assay

Total protein from cells was isolated by RIPA buffer (P0013E, Beyotime, China). Next, bicinchoninic acid (BCA) kit (G2026, Servicebio, China) was applied to assess the concentration of protein. Proteins were subjected to electrophoresis, transference onto polyvinylidene fluoride (PVDF) membranes (G6015-1, Servicebio, USA), and sealing in 5 % skimmed milk. Subsequently, membranes were probed with primary antibodies against phosphorylated-MAP1B (p-MAP1B, 1:2000, 320 kDa, PA5-23014, Invitrogen, USA), MAP1B (1:2000, 320 kDa, PA5-78052, Invitrogen, USA), Atg5 (1:1,000, 32 kDa, ab108327, Abcam, UK), light chain 3 (LC3)II/LC3I (1:2000, 14, 16 kDa, ab192890, Abcam, UK), and the endogenous control GAPDH (1:1,000, 36 kDa, ab8245, Abcam, UK) at 4 °C for 12 h. Horseradish peroxidase (HRP)-linked goat anti-rabbit secondary antibody (1:20,000, ab6721, Abcam) or goat anti-mouse secondary antibody (1:10,000, ab6789, Abcam) was then added to incubate with the membranes. BeyoECL Moon ECL reagent (P0018FS, Beyotime, China) was applied to develop the blots which were then exposed in an ImageQuant LAS 4000MINI Ultra-Sensitive Chemiluminescence Imager (Sinopharm Chemical Reagent Co. Ltd, China). Relative protein expression was represented by the ratio of target protein gray value/GAPDH gray value.

### Co-immunoprecipitation (Co-IP)

Co-IP kit (26,149, ThermoFisher, USA) was utilized to determine the interaction between MAP1B and DAPK1. In brief, total protein from HK-2 cells was extracted using IP lysis buffer. After the affinity-purified DAPK1 (PA5-17055, Invitrogen, USA) antibody was coupled to amine-based-activated resin, proteins were incubated with the resin overnight at 4 °C. Anti-IgG (#2729, Cell Signaling Technology, USA) was used as the negative control. Next, the resin was washed and the protein complexes binding to the antibody were eluted. The eluted proteins were then subjected to western blot analysis, and MAP1B was detected using a specific anti-MAP1B antibody (1:2000, 320 kDa, PA5-78052, Invitrogen, USA). The subsequent visualization using an ECL detection system was performed as described in the western blot assay.

### Statistical analysis

All results were expressed as mean±standard deviation from at least triplicate experiments, and the data analysis was carried out using Graphpad 8.0. One-way ANOVA was used to evaluate the significance among multiple groups, followed by the Bonferroni *post hoc* test. p<0.05 was identified to be statistically significant.

## Results

NTN1 knockdown facilitated apoptosis, inflammatory levels and ROS generation in LPS-treated HK-2 cells but NTN1 overexpression had the opposite effects.

**Figure 2:**
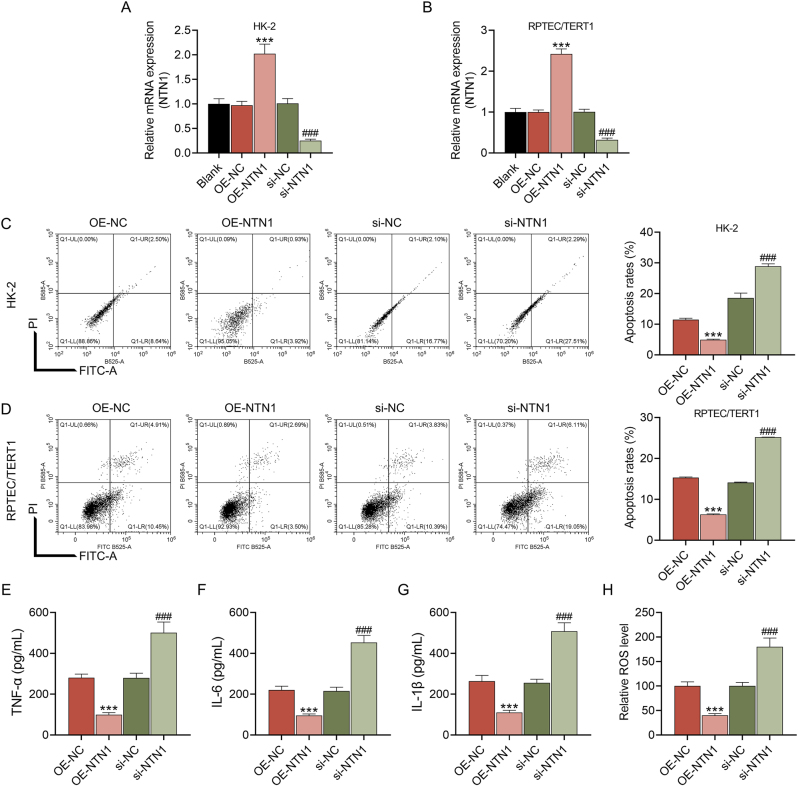
The effects of NTN1 on the apoptosis, inflammatory levels and ROS generation in LPS-treated HK-2 cells (A–B) after the transfection of NTN1 overexpression plasmid or si-NTN1, the expression of NTN1 in the blank, OE-NC, OE-NTN1, si-NC, and si-NTN1 groups was determined by qRT-PCR. GAPDH served as the internal control. (C–D) the apoptosis rate of HK-2 cells or RPTEC/TERT1 in the OE-NC, OE-NTN1, si-NC, and si-NTN1 groups was assessed by flow cytometry. (E–G) the levels of TNF-α, IL-6 and IL-1β in the OE-NC, OE-NTN1, si-NC, and si-NTN1 groups were determined by ELISA. (H) The level of ROS generation in each group was detected by DCFH-DA reagent. The data are presented as the mean±standard deviation of three independent experiments; ^***^p<0.001 vs. OE-NC; ^###^p<0.001 vs. si-NC. Abbreviation: NTN1, netrin 1; ROS, reactive oxygen species; LPS, lipopolysaccharides; OE-NTN1, NTN1 overexpression; si-NTN1, silenced NTN1; NC, negative control; qRT-PCR, quantitative real-time PCR; GAPDH, glyceraldehyde-3-phosphate dehydrogenase; TNF-α, tumor necrosis factor-α; IL-6, interleukin-6; ELISA, enzyme-linked immunosorbent assay; DCFH-DA, 2,7-Dichlorodi-hydrofluorescein diacetate.

**Figure 3:**
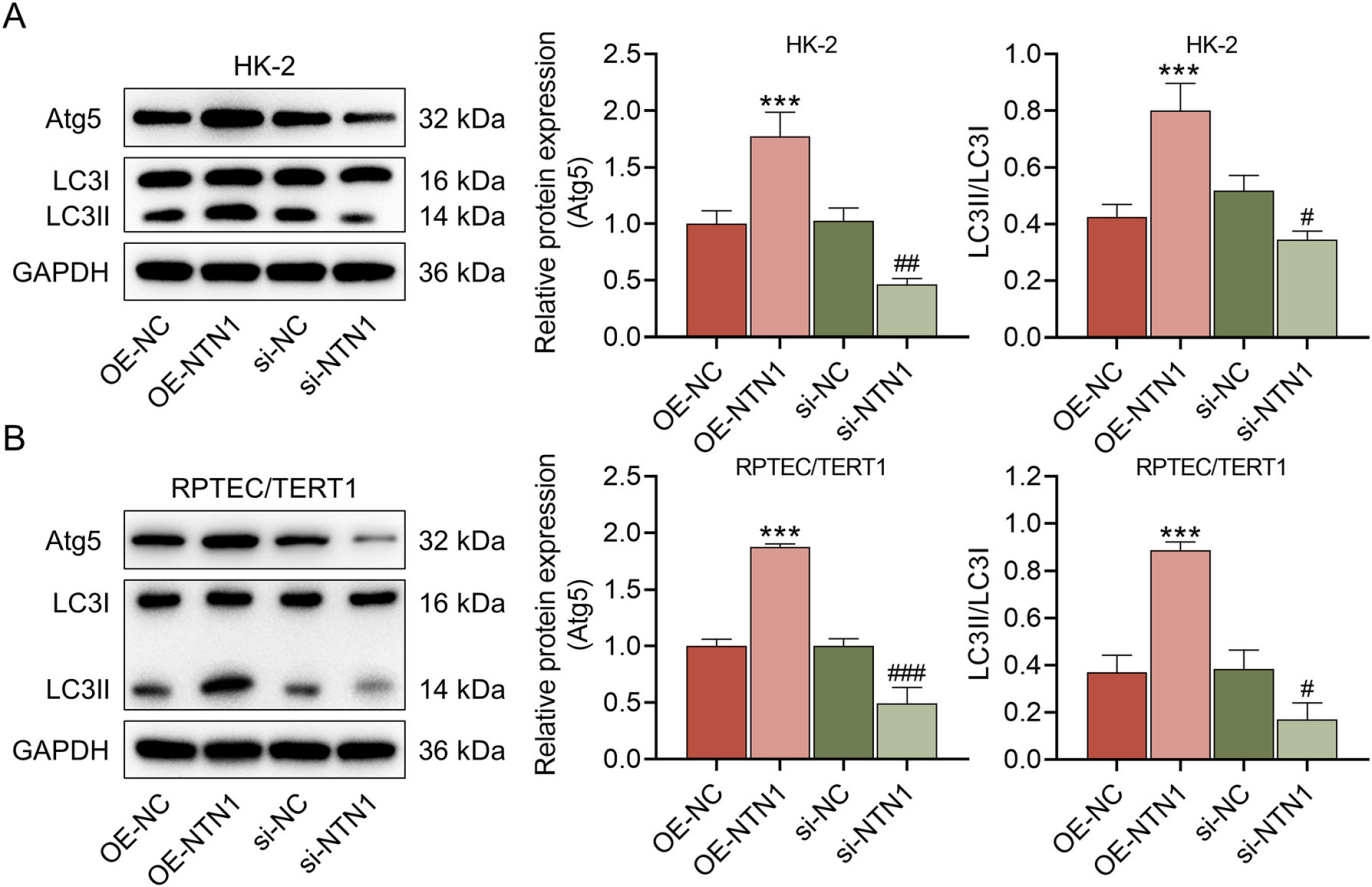
The protein expression of Atg5 and the ratio of LC3II/LC3I (A–B) the expression level of Atg5 and the ratio of LC3II/LC3I was detected by western blot, GAPDH served as the internal control. The data are presented as the mean±standard deviation of three independent experiments; ^***^p<0.001 vs. OE-NC; ^#^p<0.05, ^##^p<0.01, ^###^p<0.001 vs. si-NC.

Following transfection, it was discovered that the expression of NTN1 was prominently raised in the OE-NTN1 group and decreased in the si-NTN1 group (p<0.001, Figure 2A and B), demonstrating the effectiveness of the transfection. Additionally, the apoptosis of LPS-treated HK-2 or RPTEC/TERT1 cells was weakened by NTN1 overexpression, whereas NTN1 knockdown increased the number of apoptotic cells (p<0.001, Figure 2C and D). Besides, overexpressed NTN1 led to the downregulation of inflammatory factors (TNF-α, IL-6 and IL-1β) and ROS generation in LPS-stimulated HK-2 cells, while the above indicator levels were upregulated by NTN1 knockdown (p<0.001, Figure 2E–H). Further, the Atg5 protein expression and LC3II/LC3I level was elevated after NTN1 overexpression, but reduced after NTN1 knockdown (p<0.05, Figure 3A and B). These results unequivocally demonstrated that NTN1 mitigated LPS-induced cellular injury in renal tubular cells.

### MAP1B interacted with NTN1 and MAP1B phosphorylation inhibitor reversed the effects of NTN1 overexpression on LPS-treated HK-2 cells

**Figure 4:**
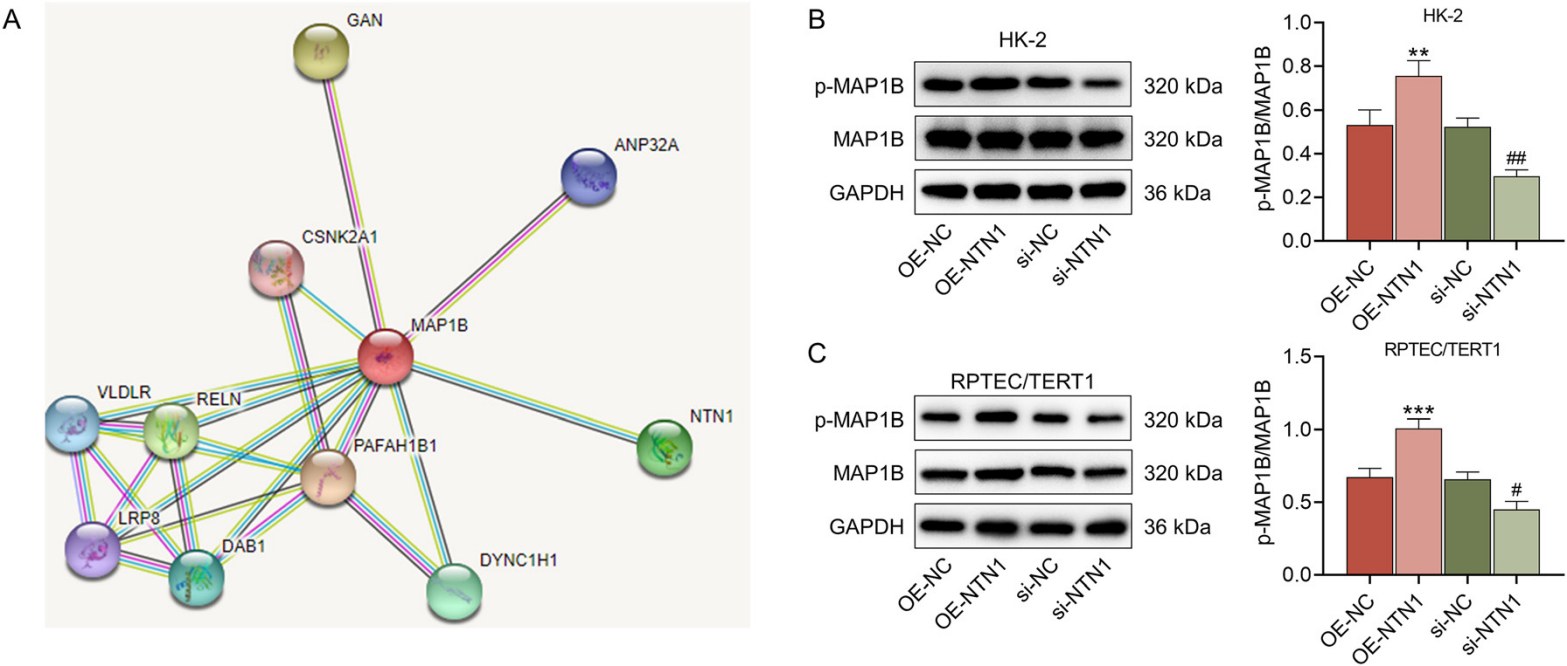
The interaction between MAP1B and NTN1 (A) the interaction between MAP1B and NTN1 was analyzed by string website (https://string-db.org/). (B–C) the ratio of p-MAP1B/MAP1B in the OE-NC, OE-NTN1, si-NC, and si-NTN1 groups was determined by western blot. GAPDH served as the internal control. The data are presented as the mean±standard deviation of three independent experiments; ^**^p<0.01, ^***^p<0.001 vs. OE-NC; ^#^p<0.05, ^##^p<0.01 vs. si-NC. Abbreviation: MAP1B, microtubule associated protein 1B; NTN1, netrin 1; OE-NTN1, NTN1 overexpression; si-NTN1, silenced NTN1; NC, negative control; GAPDH, glyceraldehyde-3-phosphate dehydrogenase.

**Figure 5:**
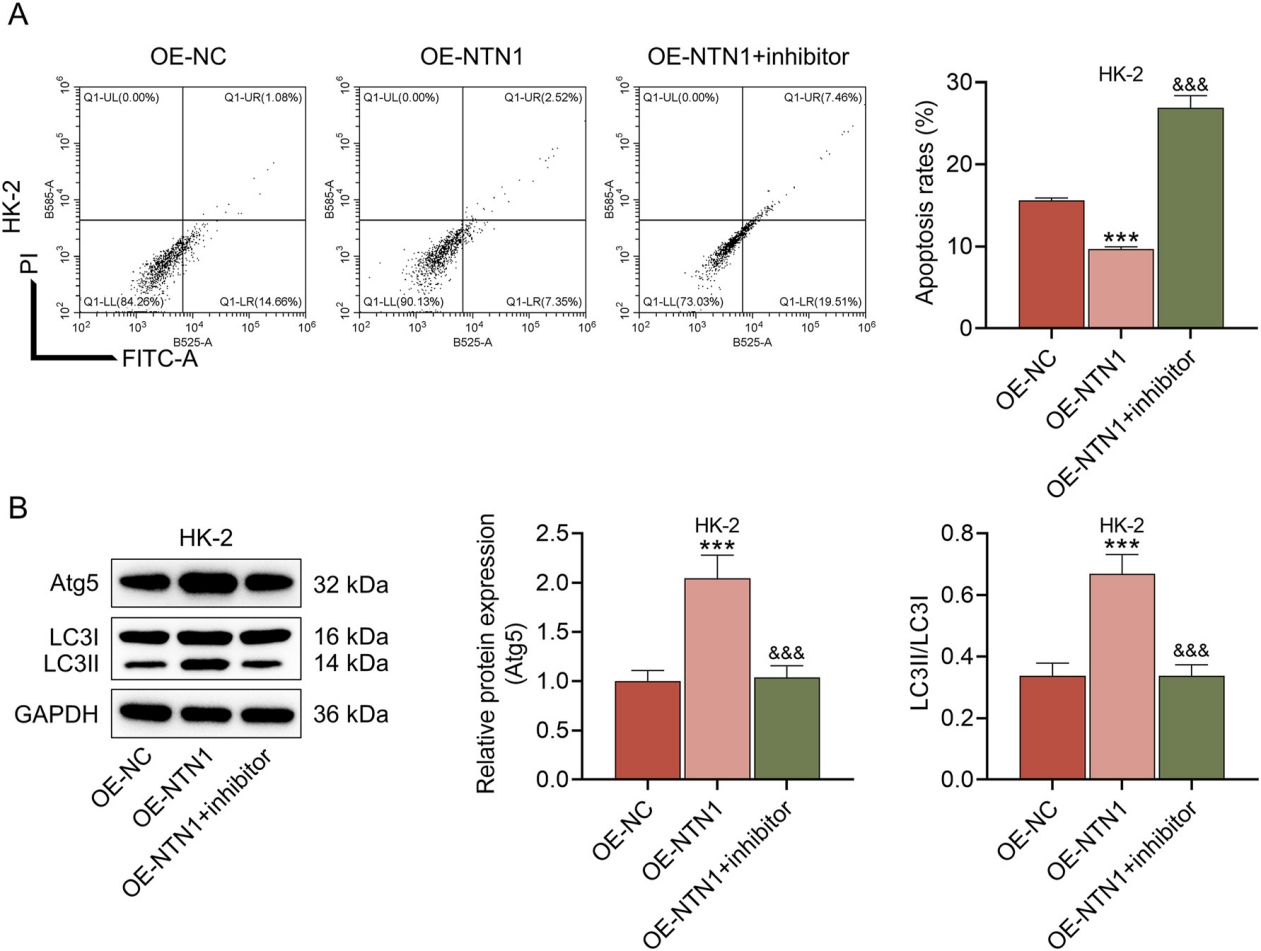
The effects of MAP1B phosphorylation inhibitor on the apoptosis and autophagy in LPS-treated HK-2 cells (A) after the treatment of MAP1B phosphorylation inhibitor, the apoptosis of HK-2 cells in the OE-NC, OE-NTN1 and OE-NTN1+inhibitor groups was evaluated by flow cytometry. (B) The protein expression of Atg5 and the ratio of LC3II/LC3I were determined by western blot. GAPDH served as the internal control. The data are presented as the mean ± standard deviation of three independent experiments; ^***^p<0.001 vs. OE-NC; ^&&&^p<0.001 vs. OE-NTN1. Abbreviation: MAP1B, microtubule associated protein 1B; LPS, lipopolysaccharides; NTN1, netrin 1; OE-NTN1, NTN1 overexpression; NC, negative control; GAPDH, glyceraldehyde-3-phosphate dehydrogenase.

To elucidate the mechanism of NTN1, we dug into its downstream mediators. According to the analysis of String website, MAP1B is an interacting protein of NTN1 (Figure 4A). Next, the interaction between MAP1B and NTN1 was further determined by Western blot, which displayed that the ratio of p-MAP1B/MAP1B was markedly elevated in the OE-NTN1 group and lowered in the si-NTN1 group (p<0.01, Figure 4B and C). To uncover the regulatory relationship between MAP1B and NTN1 in kidney injury cells, MAP1B phosphorylation inhibitor was applied to treat LPS-stimulated HK-2 cells. The results indicated that the apoptosis rate of HK-2 cells was conspicuously increased in the OE-NTN1+inhibitor relative to the OE-NTN1 group (p<0.01, Figure 5A). Moreover, MAP1B phosphorylation inhibitor diminished the Atg5 protein expression and LC3II/LC3I level of HK-2 cells that were transfected with OE-NTN1 and treated with LPS (p<0.001, Figure 5B). These data demonstrated that MAP1B phosphorylation was not merely correlated with, but was essential for NTN1-mediated cytoprotection and autophagy induction.

### MAP1B inhibited the LPS-induced injury of HK-2 cells via regulating DAPK1

**Figure 6:**
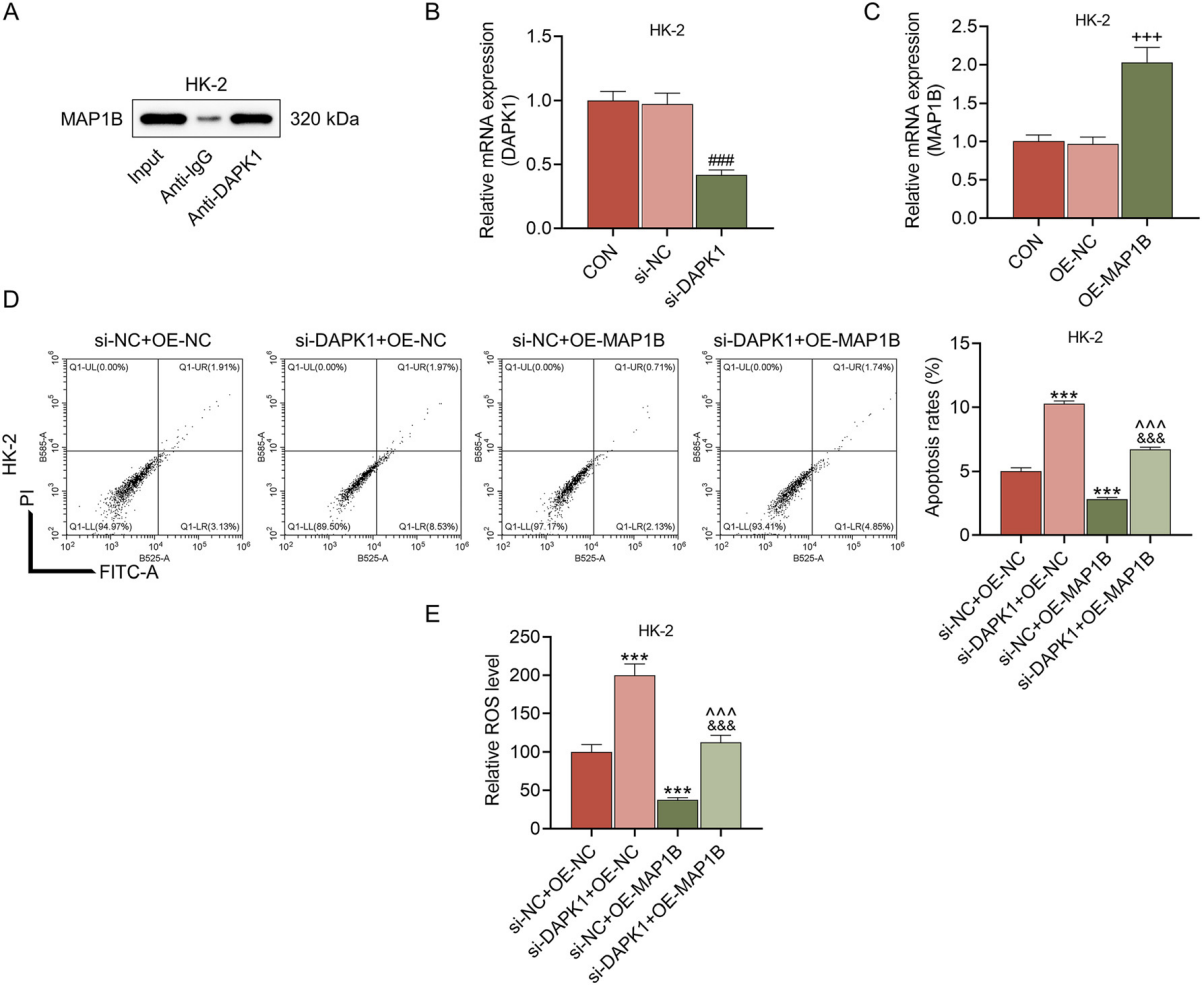
The effects of MAP1B and DAPK1 on the apoptosis and ROS generation of LPS-treated HK-2 cells (A) Co-IP assay was performed to detect the relationship between MAP1B and DAPK1. (B–C) after the transfection of si-DAPK1 (B) or OE-MAP1B (C), the mRNA expression of DAPK1 or MAP1B in HK-2 cells was tested by qRT-PCR. GAPDH served as the internal control. (D) flow cytometry was used to determine the apoptosis rate of LPS-treated HK-2 cells in the si-NC+OE-NC, si-DAPK1+OE-NC, si-NC+OE-MAP1B and si-DAPK1+OE-MAP1B groups. (E) the ROS generation in each group was detected by DCFH-DA reagent. The data are presented as the mean±standard deviation of three independent experiments; ^###^p<0.001 vs. si-NC; ^+++^p<0.001 vs. OE-NC; ^***^p<0.001 vs. si-NC+OE-NC; ^ˆˆˆ^p<0.001 vs. si-DAPK1+OE-NC; ^&&&^p<0.001 vs. si-NC+OE-MAP1B. Abbreviation: MAP1B, microtubule associated protein 1B; DAPK1, death associated protein kinase 1; ROS, reactive oxygen species; LPS, lipopolysaccharides; si-DAPK1, silenced DAPK1; NC, negative control; qRT-PCR, quantitative real-time PCR; GAPDH, glyceraldehyde-3-phosphate dehydrogenase; DCFH-DA, 2,7-Dichlorodi-hydrofluorescein diacetate.

**Figure 7:**
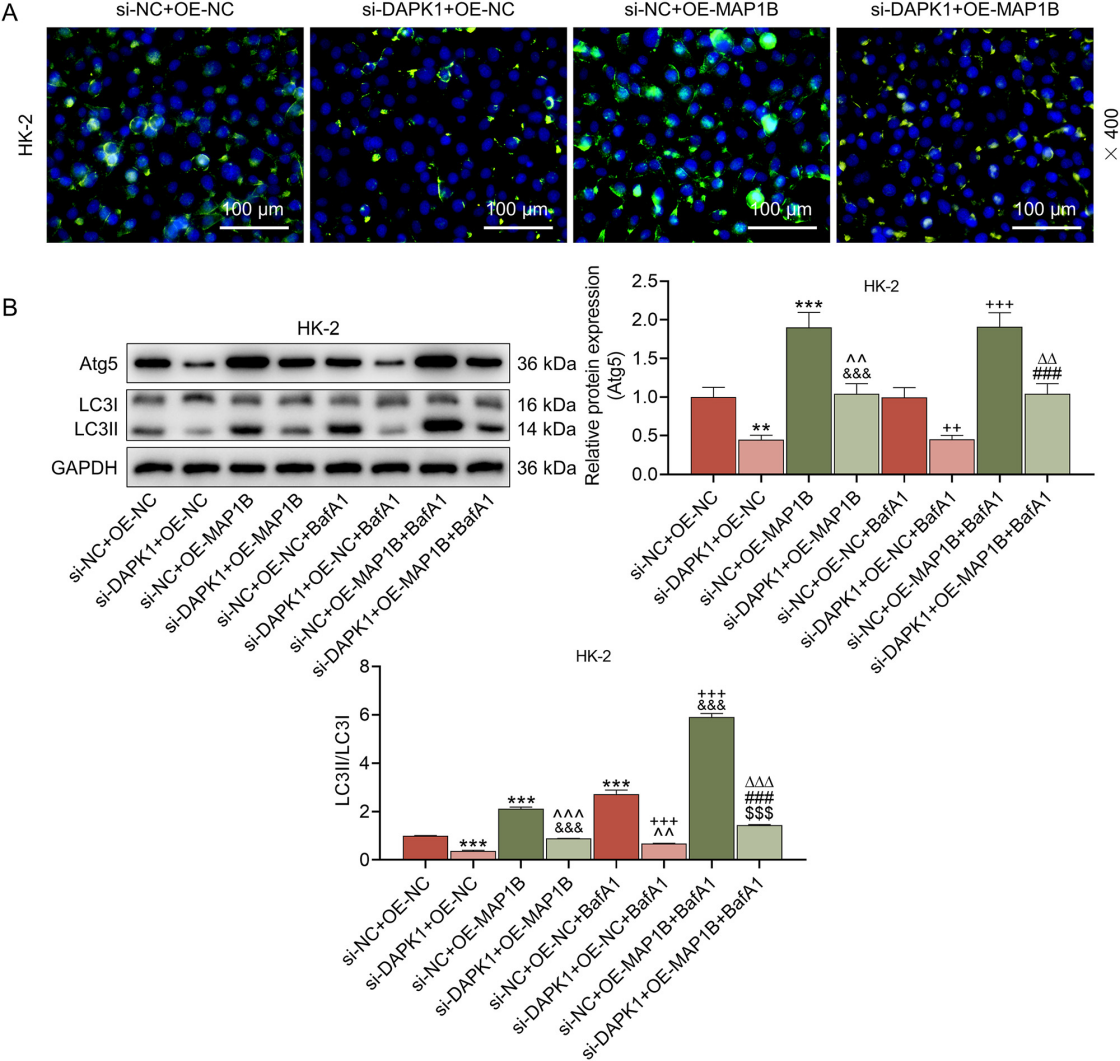
The effects of MAP1B and DAPK1 on the membrane blebbing and autophagy of LPS-treated HK-2 cells (A) the membrane blebbing of LPS-treated HK-2 cells in the si-NC+OE-NC, si-DAPK1+OE-NC, si-NC+OE-MAP1B and si-DAPK1+OE-MAP1B groups was observed under the microscope (magnification × 400, scale bar=100 μm). (B) The protein expression of Atg5 and the ratio of LC3II/LC3I in each group were determined by western blot. GAPDH served as the internal control. The data are presented as the mean±standard deviation of three independent experiments; ^**^p<0.01, ^***^p<0.001 vs. si-NC+OE-NC; ^ˆˆ^p<0.01, ^ˆˆˆ^p<0.001 vs. si-DAPK1+OE-NC; ^&&&^p<0.001 vs. si-NC+OE-MAP1B; ^++^p<0.01, ^+++^p<0.001 vs si-NC+OE-NC+BafA1; ^ΔΔ^ *p*<0.01, ^ΔΔΔ^ *p*<0.001 vs. si-DAPK1+OE-NC+BafA1; ^###^p<0.001 vs. si-NC+OE-MAP1B+BafA1; ^$$$^ p<0.001vs. si-DAPK1+OE-MAP1B. Abbreviation: MAP1B, microtubule associated protein 1B; DAPK1, death associated protein kinase 1; LPS, lipopolysaccharides; si-DAPK1, silenced DAPK1; NC, negative control; GAPDH, glyceraldehyde-3-phosphate dehydrogenase.

Given that MAP1B is a microtubule-associated protein and a convergence point of the cytoskeleton [30], and autophagy is a membrane-driven process [31], we hypothesized that MAP1B might interact with other regulators, thereby mechanistically linking the cytoskeleton to autophagy. Subsequently, the results of Co-IP assay presented that there was an interplay between MAP1B and DAPK1 (Figure 6A). To define the functional hierarchy within this MAP1B-DAPK1 complex, we performed a rescue experiment. We transfected si-DAPK1 and MAP1B overexpression plasmid into HK-2 cells, and found that the mRNA expression of DAPK1 was decreased in the si-DAPK1 group and that of MAP1B was raised in the OE-MAP1B group (p<0.001, Figure 6B and C). It was observable that the levels of apoptosis rate and ROS were upregulated by DAPK1 silencing and downregulated by MAP1B overexpression (p<0.01, Figure 6D and E), and the levels in the si-DAPK1+OE-MAP1B group was distinctly lower than those in the si-DAPK1+OE-NC group and higher than those in the si-NC+OE-MAP1B group (p<0.001, Figure 6D and E). In addition, MAP1B overexpression promoted, while DAPK1 silencing suppressed the membrane blebbing of HK-2 cells (Figure 7A). In the meanwhile, MAP1B overexpression reversed the inhibiting effects of DAPK1 silencing on the membrane blebbing (Figure 7A). Besides, knockdown of DAPK1 markedly reduced the LC3-II/LC3-I ratio under both normal (si-NC+OE-NC) and BafA1-treated conditions (p<0.001, Figure 7B). The level of Atg5 and LC3-II/LC3-I ratio were higher in si-NC+OE-MAP1B group than si-NC+OE-NC group. Crucially, in the presence of BafA1, MAP1B overexpression led to a substantial accumulation of LC3-II and increased Atg5 level, which was more prominent than those in the si-NC+OE-NC+BafA1 group (p<0.001, Figure 7B). In cells with DAPK1 knockdown (si-NC+OE-MAP1B vs. si-DAPK1+OE-MAP1B group or si-NC+OE-MAP1B+BafA1 vs. si-DAPK1+OE-MAP1B+BafA1 group), the ability of MAP1B to increase the LC3-II/LC3-I ratio was severely compromised (p<0.001, Figure 7B). The above findings corroborated that the ability of MAP1B to enhance autophagic flux was negated in the absence of DAPK1.

## Discussion

Existing research proved that endotoxin-mediated renal tubular epithelial cell injury is an important pathological mechanism of S-AKI [32]. During the progression of S-AKI, the release of soluble inflammatory mediator LPS is increased, which promotes the ROS generation and the expressions of multiple inflammatory factors (TNF-α, IL-6, and IL-1β), ultimately inducing the apoptosis of renal tubular epithelial cells [33], 34]. In this study, HK-2 cells were transfected with NTN1 overexpression plasmid or si-NTN1 and then stimulated with LPS to investigate the effect of NTN1 on S-AKI model cells. The experimental results showed that NTN1 upregulation inhibited the apoptosis, inflammation and ROS generation in LPS-treated HK-2 cells, whereas NTN1 knockdown displayed the opposite effects, indicating that NTN1 could alleviate the renal tubular epithelial cell injury.

Next, we found that MAP1B is an interacting protein of NTN1 through bioinformatics prediction. Reportedly, MAP1B may be a downstream effector in the NTN1 signaling pathway, and NTN1 can regulate MAP1B phosphorylation *in vivo* and *in vitro* [35]. Consistent with the previous study, we found that NTN1 upregulation elevated p-MAP1B level. MAP1B belongs to MAPs family expressed in the nervous system development [36]. MAP1B and its phosphorylated form p-MAP1B can induce cytoskeleton rearrangement and promote axon growth and regeneration by regulating actin and microtubules [36], 37]. Previous research has not pinpointed the effect of MAP1B on S-AKI, while we found that MAP1B phosphorylation inhibitor reversed the anti-apoptosis effect of NTN1 on LPS-treated HK-2 cells.

There is a report suggesting that MAP1B phosphorylation may play an important role in DAPK-induced membrane blebbing [25] and MAP1B is a key target of DAPK1 [29]. Our study further verified through Co-IP assay that MAP1B could interact with DAPK1. DAPK is a gene directly related to apoptosis, and its protein product is a serine/threonine protein kinase with a molecular weight of 160 kDa that is closely associated with the cytoskeleton and regulated by calcium and calmodulin [38]. DAPK is a protein that can mediate cell death in response to interferon-γ (IFN-γ), and DAPK is a key molecule shared among multiple cell death signaling pathways and can mediate cell death induced by various upstream signals including TNF-α, TGF-β, and CD95 [39], 40]. In addition to apoptosis, DAPK is also implicated in cell progression such as autophagy and anoikis [41], 42]. Ben Harrison et al. unveiled that DAPK-1 can co-express with MAP1B to form an interaction, which can synergistically induce membrane blebbing and stimulate autophagy [29]. Further, the segregation of these vesicle compartments on microtubules is regulated, in part, by a signal transduction pathway, in which DAPK1 phosphorylates MAP1B, a process maintained by E3 ubiquitin ligase Mindbomb 1 (MIB1) [43]. Activated DAPK1 phosphorylates Pellino1 at Ser39, which triggers polyubiquitination and turnover of Pellino1, leading to TRIF-RIP1 signalosome release for caspase-8 recruitment and thereby coupling the LPS/MyD88-dependent inflammatory response to tubular damage in S-AKI [44]. In this study, we observed that DAPK1 knockdown promoted cell apoptosis and ROS generation while suppressing membrane blebbing, which was significantly reversed by MAP1B overexpression, suggesting a functional hierarchy wherein MAP1B acts upstream of or in parallel to DAPK1 to coordinate these cellular processes, including blebbing.

Autophagy, a highly conservative physiological process in which lysosomes phagocytize proteins or organelles in cells, can provide cells with raw materials under physiological conditions, so as to meet their metabolic needs and renew some organelles [45]. However, under pathological conditions, the abnormal autophagy can promote cell apoptosis [46]. Recent studies presented that autophagy plays an important role in the process of S-AKI and LPS can induce apoptosis of renal tubular epithelial cells [47]; meanwhile, the level of autophagy in cells fluctuates, showing a trend of first increasing and then decreasing [48], 49]. In addition, Shuqin Mei et al. found that autophagy can inhibit apoptosis and protect renal tubular epithelial cells from LPS-mediated cell injury [50]. In our study, we discovered that overexpressed NTN1 increased the levels of Atg5 protein expression and LC3II/LC3I, and these changes were reversed by MAP1B phosphorylation inhibitor. To clarify the mechanistic implications of these findings, we explored the specific roles of these proteins in the autophagy cascade. The autophagy-related protein Atg5 is essential during the initial stages of autophagosome formation, particularly in mediating the lipidation of LC3 [51]. The conversion of soluble LC3-I to the lipidated, autophagosome-membrane-bound LC3-II form, reflected by an increased LC3-II/LC3-I ratio, is a well-established hallmark of ongoing autophagosome biogenesis and a key indicator of autophagy [52]. Therefore, NTN1-induced upregulation of both Atg5 and the LC3-II/LC3-I ratio provided strong molecular evidence that NTN1 activated the autophagic process at the initiation and elongation stages. The effect of NTN1 was reversed by MAP1B phosphorylation inhibitor, which provided additional support that a functional cooperation existed between MAP1B and NTN1 to impact autophagy in LPS-treated HK-2 cells.

Interestingly, previous studies showed that overexpression of DAPK can lead to membrane blebbing and the appearance of autophagic vesicles, while its co-expression with MAP1B results in the disruption of microtubules, the induction of membrane blebbing and concomitant autophagic vesicle formation. Of note, blebbing could be inhibited by treatment with the autophagy inhibitor 3-methyladenine [25]. DAPK1-induced membrane blebbing is known to involve the regulation of actomyosin contractility [25]. Existing studies have shown that inhibition of RHO-related kinase (ROCK), a key downstream effector of cytoskeletal reorganization, can effectively suppress membrane blebbing [53]. This is particularly relevant in the context of S-AKI, where the Rho-associated kinase inhibitor Fasudil demonstrates significant anti-inflammatory, antioxidant, and nephroprotective effects, primarily by repressing STAT-3 and NLRP-3 signaling [54]. Furthermore, ROCK2 knockdown can alleviate LPS-induced HK-2 cell injury via inactivation of the NF-kB/NLRP3 signaling pathway [55]. Therefore, membrane blebbing formation in S-AKI may be a “dynamic indicator of cellular stress response”: on the one hand, it exerts a protective effect in synergy with autophagy, and promotes pathological damage with inflammation and out-of-control skeletal disorders; on the other hand, its regulatory pathways (such as ROCK) also provide interventional targets for the treatment of S-AKI. Accordingly, more experiments are needed for further verification.

There are some limitations in this study. First, while this study delineates a novel downstream mechanism by which NTN1 confers protection in S-AKI, the upstream signaling that induces NTN1 expression in response to septic insult remains an open question. Future investigations are warranted to elucidate this initial trigger. Moreover, a primary limitation lies in the reliance on *in vitro* data from a human renal tubular epithelial cell line. While the cell model is crucial for delineating the precise molecular details of the NTN1-MAP1B-DAPK1 axis, future validation in more physiologically complex systems is essential, such as the use of a murine S-AKI model with kidney-specific adeno-associated virus (AAV)-mediated gene modulation of NTN1 to establish causal relationships *in vivo*, or the measurement of NTN1 levels in biofluids and pathway component expressions in clinical kidney tissues from S-AKI patients.

To conclude, NTN1 promotes cell membrane blebbing to induce autophagy in LPS-treated HK-2 cells *in vitro* by regulating MAP1B and DAPK1. This discovery deepen our understanding on the potential of NTN1 as a target for treating S-AKI. The *in vivo* and clinical validation in the future, as outlined above, will contribute to translating these mechanistic insights into clinical benefits.
